# Effect of Simultaneous Dietary Supplementation of Betaine, Selenomethionine, and Vitamins E and C under Summer Conditions in Growing–Finishing Pigs

**DOI:** 10.3390/vetsci11030110

**Published:** 2024-03-01

**Authors:** Lotte De Prekel, Dominiek Maes, Alice Van den Broeke, Bart Ampe, Marijke Aluwé

**Affiliations:** 1 Unit of Porcine Health Management, Department of Internal Medicine, Reproduction and Population Medicine, Faculty of Veterinary Medicine, Ghent University, 9000 Ghent, Belgium; 2 Fisheries and Food (ILVO), Flanders Research Institute for Agriculture, 9820 Merelbeke, Belgium

**Keywords:** precision livestock farming, respiration rate, rectal temperature, meat quality, temperature–humidity index

## Abstract

**Simple Summary:**

Due to climate change, heat waves occur more often, and the annual average temperature increases, which may contribute to the negative effects of heat stress in pigs. This study explored ways to help growing–finishing pigs cope with heat stress during hot weather. Two groups of pigs were given different diets—one standard and one enriched with specific feed additives. The pigs’ respiration rates, rectal and skin temperatures, performances, and meat qualities were monitored. During a heat wave, the group with the enriched diet did not need as much drinking water compared to the standard diet group. Both groups, without distinguishing between dietary treatments, showed increased respiration rates and skin temperatures during higher heat loads. The findings suggest that the enriched diet may help reduce the need to increase daily water intake during hot periods. Furthermore, this study highlighted the sensitivity of the parameter of respiration rate in pigs suffering from (mild) heat stress.

**Abstract:**

Heat stress in pigs negatively affects welfare, health, and performance. Osmoprotectants and antioxidants may alleviate oxidative damage during hot periods. We investigated whether an additive-enriched feed can reduce negative effects in pigs during summer conditions. Sixty growing–finishing pigs were allocated into two groups: the control (CF) and summer feed (SF) group. The CF group contained 0.4 mg/kg inorganic selenium and 100 ppm vitamin E, while the SF group contained 0.3 mg/kg inorganic selenium, 0.1 mg/kg selenomethionine, 200 ppm vitamins E and C, and 0.2% betaine. Feed was offered ad libitum. Respiration rate, rectal and skin temperature, behaviour, and weight gain were assessed weekly. Daily measurements of these parameters were performed during a 3-day heat wave (temperature humidity index (THI) ≥ 75) and during an artificial heating period. Individual average daily water intake (ADWI) and feed intake were measured using RFID systems. The ADWI of the SF group did not change during heat load while it increased for the CF group. Independent of dietary treatment, increases in ∆THI or the THI were accompanied by significant increases in both respiration rate and skin temperature. In conclusion, the SF may induce a reduced need to increase ADWI during hot periods. In addition, mainly skin temperatures and especially respiration rates revealed the sensitivity of pigs to heat stress.

## 1. Introduction

Heat stress (HS) has become a key issue in livestock farming, as it directly impacts animal welfare, health, and livestock performance [1]. In the last decade, Belgium faced more heat waves, and the average annual temperature increased by 5.2 °C, compared to 1879 [2]. Under predicted climate change scenarios, the average annual temperature in Belgium will increase by 0.7 °C to 5 °C by the end of the century [3,4], exacerbating the effects of HS in pigs. Compared to other farm animals, pigs are more sensitive to increases in ambient temperature [5]. For heat loss, pigs mainly rely on respiration, as they have very few functional sweat glands and a non-vascularized subcutaneous adipose tissue layer, which makes heat loss from the skin surface even more difficult [6]. As a result, heat-stressed pigs show changes in physiological parameters, such as an increased core temperature and respiration rate (RR) [7,8], and welfare parameters, such as behavioural changes [5,9].

Heat stress can also impair performance. Decreased feed intake is the primary reported consequence of HS in animals to reduce heat production [10,11,12]. Reduced feed intake is partly responsible for decreases in average daily gain (ADG) during periods of high heat load [8,13]. Under intense heat load conditions, feed efficiency will therefore also decrease due to insufficient nutrient intake [14]. Additionally, during periods of intense heat, the body changes the hierarchy of nutrient utilisation in an attempt to maintain euthermia [15]. This can cause osmotic stress, hypoxia, increased free radical damage, and compromised integrity of the gastrointestinal tract, resulting in impaired permeability to macromolecules [16,17]. These direct and indirect effects of HS may impair digestion and inhibit adequate nutrient utilisation.

Some feeding strategies show the potential to mitigate the negative effects of HS. These feeding strategies may include alterations in the chemical composition of the diet [18,19] or the addition of functional feed additives. Antioxidants are frequently used as feed additives to mitigate the effects of HS in different animal species, as they prevent osmotic stress caused by free radicals and oxidants. Non-enzymatic antioxidants, such as vitamin E and vitamin C, can improve the efficiency of the antioxidant system when used with the synergetic cooperation of enzymatic antioxidants, such as glutathione peroxidase. Selenomethionine plays an important role in this process [17]. Betaine, which has been tested extensively in pigs and poultry during thermoneutral conditions, functions as an osmoprotectant and methyl group donor and thus may also reduce the adverse effects of HS [20,21]. Furthermore, the addition of some of these feed additives can positively influence carcass traits and pork meat quality [20,21,22,23,24,25,26].

The supplementation of individual additives like betaine, selenomethionine, vitamin E or vitamin C has already been investigated under thermoneutral conditions, but the effects of concurrent supplementation of different additives are less clear [17]. In addition, the effect of selenium during high heat load periods has been studied in poultry [27] but less in growing–finishing pigs. A larger number of reports on betaine supplementation in poultry during high heat loads [20] is available in comparison to similar studies on pigs. Various studies also supplied additives outside of the regulatory limits, limiting their applicability for practical use. To our knowledge, the combination of vitamin E, vitamin C, selenomethionine, and betaine in feed as a measure to combat heat stress in growing–finishing pigs has not yet been tested. 

The aim of the present study was to investigate the effect of dietary supplementation of betaine, vitamin E, vitamin C, and selenomethionine in growing–finishing pigs under summer conditions. Concentrations of the tested additives were within legal limits. Physiological parameters, animal welfare, carcass and meat quality, and individual performance parameters were evaluated to determine the possible effects of dietary supplementation on symptoms of heat stress. 

## 2. Material and Methods

### 2.1. Experimental Setup and Animals

**Figure 1:**
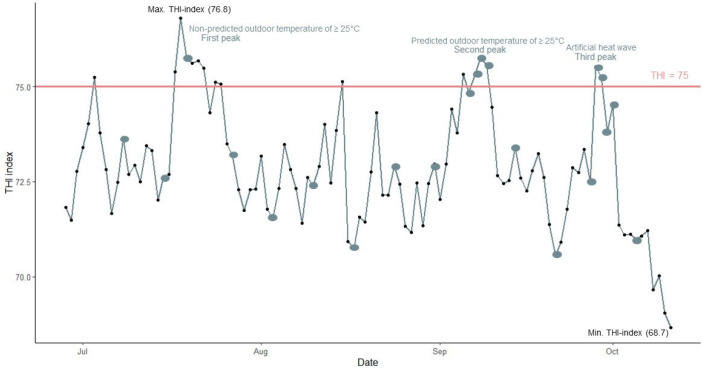
Evolution of the daily maximum temperature–humidity index (THI) and indication of the three heat peaks and min (68.7) and max (76.8) THI during the entire growing–finishing period. (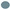 = days where physiological parameters and animal behaviour were observed between 13:00 and 17:00; 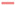 = a THI of 75 indicates a warning for heat stress).

A total of 60 mixed-sex growing–finishing pigs (hybrid sow × Piétrain) were divided into a control (CF) and a summer feed (SF) group, with two pens of 15 animals per treatment. The distribution was made so that each pen had about the same average weight and an equal distribution of barrows (castrated boars) and gilts. The diets were provided in two phases: a starter diet and a grower diet starting at 15 weeks of age. In both phases, SF was enriched with selenomethionine, betaine, vitamin C, and vitamin E (Table 1). Additive levels were implemented based on (1) literature data about the effectiveness of feed additives as a measure against heat stress [17,20,28,29,30,31,32,33,34], and (2) the advice of the feed expert group of the research project Coolpigs. Feed and water were provided ad libitum to both groups.The trial started on 30 June 2021 at 10 weeks of age (31.0 ± 2.9 kg) and ended on 12 October 2021 at 25 weeks of age and when the pigs had reached slaughter weight (120.6 ± 11.3 kg). When the outside temperature was predicted to exceed 25 °C for a minimum of three consecutive days, physiological parameters and animal behaviour were observed more intensely (Figure 1). Additionally, an artificial heat wave was induced for three days in the last week of September when the animals were 23 weeks old. Three pigs were removed from the trial: one due to lameness and two due to stress from having been stuck in the feeding system. All three came from the same pen (CF1) and were not included in the data set for statistical analysis.

#### 2.1.1. Housing

**Figure 2:**
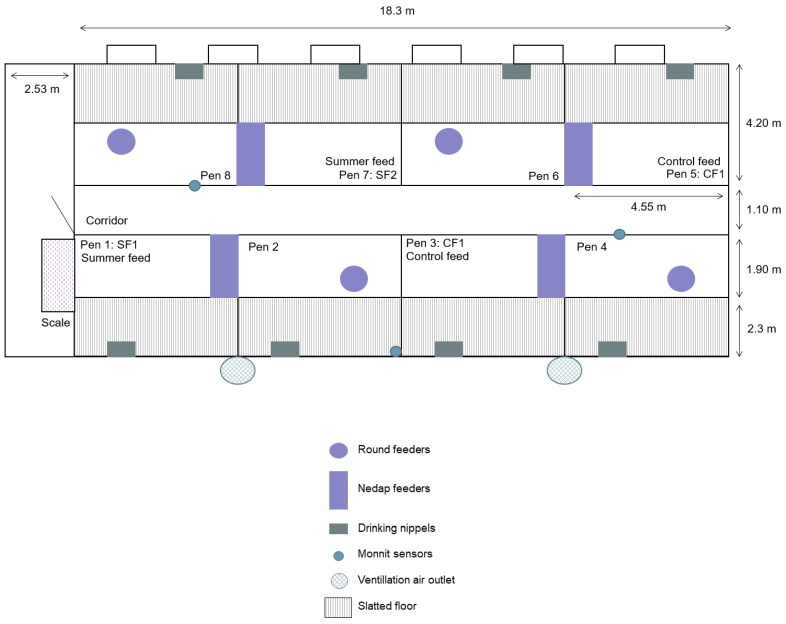
Schematic overview of the entire compartment.

The trial was performed at ILVO’s experimental farm (Melle, Belgium). The stable is comprised of eight pens (Figure 2), four of which were used in the trial. A Nedap feeding system (Nedap Prosense©, Nedap N.V., The Netherlands) was located in the right front corner of each pen with one drinking nipple in the centre back of the pen. The other four pens were not used in this trial as they were not equipped with a Nedap system. Those pens were populated with pigs kept according to standard farm management. All pens in the compartment had a partially slatted floor with a total pen surface of 19.11 m^2^ (1.27 m^2^/animal). The compartment was artificially lit from 08:00 to 16:30 plus natural light from one window (90 × 80 cm) on the left side (southwest) of the compartment and six windows (90 × 60 cm each) on the right (north–east). The air inlet for the mechanical ventilation system (110 × 35 cm) was located under the six windows.

#### 2.1.2. Climate Control

The stable climate was automatically controlled with a climate computer (Hotraco Agri©, Hotraco Group, The Netherlands) during the trial. The two extra heating devices used to create an artificial heat wave (Thermobile ITA-45 Robust™, Thermobile, The Netherlands) had a heating power of 45.1 kW and an air displacement of 3000 m^3^ via a heating duct (6 m, Ø 0.4 m). During the artificial heat wave, the temperature was kept constant at approximately 31 °C between 07:00 and 22:00. Between 22:00 and 07:00, the temperature was reduced to 26 °C. These limits were based on temperatures achieved in the stable in the summer of 2020 during a naturally occurring heat wave. Two climate sensors (Monnit^®^, Monnit Corporation, South Salt Lake, UT, USA) were placed in the corridor at a height of 125 cm. Two additional sensors were placed in the back of the pens in the middle of the compartment at a height of 110 cm (Figure 2). These sensors logged the relative humidity and ambient temperature in the stable every two hours for the entire trial period. During the artificial heat wave, four additional sensors (HOBO onset^®^, Bourne, MA, USA) were placed at the pens of SF1, SF2, CF1, and CF2 in the corridor at a height of approximately 150 cm. They measured relative humidity and temperature every five minutes. The Monnit sensors were used for analysis.

#### 2.1.3. Temperature–Humidity Index

Depending on the measured parameter and relative humidity, pigs can start showing signs of heat stress at different temperatures [8]. To monitor heat stress correctly, the temperature–humidity index (THI) was used, which combines ambient temperature and relative humidity (1). Temperature–humidity index values in excess of certain limits indicate a risk of HS in animals. The THI limits for potential HS in this trial were based on the behaviour of pigs during handling and transport, i.e., 75 ≥ THI > 79: warning for HS, 79 ≥ THI > 84: danger for HS, THI ≥ 84: great danger for HS [35,36,37,38].

(1) $$\begin{array}{c} THI=0.72\times T_{DB}+0.72\times T_{WB}+40.6\\ T_{WB}=T_{DB}\times {tan}^{-1}\left(0.151977\times \sqrt[{2}]{\left(RH+8.313659\right)}\right)+{tan}^{-1}\left(T_{DB}+RH\right)–{tan}^{-1}\left(RH–1.676331\right)+0.00391838\times {RH}^{\frac{3}{2}}\times {tan}^{-1}(0.023101\times RH)–4.686035 \end{array}$$

where THI: temperature–humidity index, T_DB_: dry-bulb temperature [°C], T_WB_: wet-bulb temperature [°C], and RH: relative humidity [%].

### 2.2. Measurements at Farm Level

#### 2.2.1. Physiological Parameters and Animal Behaviour

Physiological parameters monitored were respiration rate (RR), rectal temperature (T_rectal_), and skin temperature (T_skin_). Per pen, seven randomly selected reference animals were chosen for physiological measurements. The reference animals did not change during the entire trial. Animal behaviour was measured at pen level. The parameters were measured weekly during the entire trial period. In addition, the parameters were evaluated daily during a predicted natural heat wave (three consecutive days ≥ 25 °C). During the artificial heat wave, the parameters were measured during the three days of heating, one day before and one day after heating. The observations always started at 13:00 h and were conducted by the same group of observers. 

Respiration rate (breaths per min) was scored visually based on the number of flank movements per 30 s multiplied by two. Respiration rate was only evaluated when a pig was lying down in a resting position. Rectal temperature (°C) was measured using a digital thermometer (Veterinär-thermometer SC12, Scala electronics GmbH, Germany) for approximately 15 s after the pigs were moved to a smaller pen located in the corridor. Skin temperature (°C) was measured using an infrared camera (Testo 875-i2™, Testo, The Netherlands), with the camera focused on the entire flank of the pig. The emission coefficient was set to ε = 0.98, which is the value for biological tissues [39,40]. The images were analysed afterwards using the Testo software, calculating the average T_skin_ of a manually indicated, oval area of the pig’s flank as described by Brown-Brandl et al. [39]. Behaviour was observed via scan sampling. Observations started with a first pen, where each animal was categorised according to the type of activity. The ethogram based on the method of Ekkel et al. [41] was used for the observations (Table 2). The behavioural assessment was then continued in the other pens. After evaluating all pens, the observer waited three minutes before starting with the first pen again. In total, the behaviour was evaluated eight times per pen during a period of approximately 30–60 min. Afterwards, the average of all the behaviour results was calculated and converted into a percentage.

#### 2.2.2. Correlations

A correlation coefficient between T_rectal_ and T_skin_ was calculated in the second heat period and during the entire growing–finishing period to verify the reliability of T_skin_ as non-invasive indicator of HS. Also, the correlation with ambient temperature was taken into account to check the reliability of T_skin_ as a physiological parameter.

#### 2.2.3. Performance Parameters

Pigs were individually tagged with a low-frequency Radio Frequency Identification (RFID) ear tag. The feeding system (Nedap Prosense, Nedap livestock management©, Groenlo, The Netherlands) registered every animal’s visit to the feeder and the individual’s number and feed intake. In addition, all pigs were weighed individually 1 ×/week. Based on this data average daily feed intake (ADFI), average daily gain and feed conversion ratio were calculated. Each pig was also tagged with a high-frequency tag to register the drinking pattern at the nipple. Animal number, daily drinking time, number of drinking visits, and average daily water intake (ADWI) were registered as described by Maselyne et al. [42,43]. Drinking time was measured by the time of the same pig’s first and last registered visit. Registrations shorter than three seconds (too short to drink) or longer than 180 s (too long to drink; probably lying or exploring in front of the nipple) were removed from the dataset. The daily water intake was measured with a flow meter (FT210-Turboflow, Gems Sensors and Controls Inc., Plainvilles, CT, USA) installed in front of the drinking nipple that registered the flow during each drinking visit. The pigs could partly regulate the flow rate by biting down more or less forcefully on the nipple.

### 2.3. Measurements in the Slaughterhouse

The measurements of observations in the lairage area, carcass traits and meat quality (instrumental and sensory) are described in Supplementary Material and Methods S1.

### 2.4. Statistical Analysis

Statistics were calculated in R^®^ software (version 4.1.1) (RStudio, R Core Team, Auckland, New Zealand), where the effect of the diet, ∆THI, and the interaction of diet and ∆THI were evaluated based on the independent physiological parameters (RR, T_rectal_ and T_skin_) and animal behaviour. Interaction terms of the fixed effect with *p*-values > 0.05 were excluded from the final model. Differences were considered significant if *p* ≤ 0.05. A broken line model based on the collected data with a pre-set cut-off THI value of 75 (as based on the limit of potential signs of HS by NWSCR [38]) was used to determine the slope of the animal parameters of the diet groups under greater ∆THI. All THI values lower than 75 had ∆THI = 0, while THI values higher than 75 had a ∆THI = 75-THI. When the data did not fit the broken line model, the data were fitted with the THI increase (no pre-set cut-off) instead of ∆THI. In the model, age of the pigs was used as co-variable. Observation date and pig ID within the pen were used as random variables. The effect of the diet, the period of HS (before, during, and after), and the interaction of diet x period on average daily water intake, daily drinking visits, drinking time, and average daily feed intake were determined using linear mixed model. This was established by evaluating these parameters before, during, and after a heat peak totalling 16 days (number of days within the three peaks; Figure 1). The same number of days before and after a heat peak were implemented in the analysis. In the model, the heat peak (first, second, or third peak) was used as co-variable, and pig ID within the pen was used as random variable. A post hoc was performed when significant differences were found. The pen was considered an experimental unit.

## 3. Results

### 3.1. Temperature Humidity Index

The average maximum daily THI during the trial was 72.9 (Figure 1). Only 16 days had a maximum daily THI above 75; the highest maximum daily THI was 76.8. The first THI peak above 75 (17 to 25 July) was not forecast as a period of at least three days at ≥25 °C. During that period, performance parameters were registered, but daily observations of physiological parameters were not performed. The second peak (5 to 9 September) had been accurately forecast, so four daily observations were implemented. Of these, only three observation days met the requirement of a maximum daily THI ≥ 75. The last small peak occurred when the artificial heat wave was induced from 28 to 30 September, where only two observation days had a maximum daily THI ≥ 75. In total, there were 21 observation days, of which only six took place when the maximum daily THI was ≥75. The average maximum daily THI index of the three heat peaks was 75.4.

### 3.2. Measurements at Farm Level

#### 3.2.1. Physiological Parameters and Animal Behaviour

There was no effect of diet or diet × ∆THI interaction on the RR and T_rectal_ of the growing–finishing pigs (Table 3). A significant effect (*p* < 0.001) of ∆THI on RR was found, where the RRs for both feed groups increased with an increasing THI (34.5 breaths per min, per ∆THI above the baseline of THI = 75). 

The broken line model was not used for T_skin_ due to the linear progression of the rising T_skin_ with an increasing THI, where no clear inflexion point could be seen. Also, no effect of diet or the diet × THI interaction on T_skin_ was found (Table 3). Nevertheless, the THI had a significant effect (*p* < 0.001) on T_skin_, as the T_skin_ for both groups increased when the THI also increased (0.294 °C per THI).

No significant diet effect or diet × ∆THI interaction was found on the different behaviour parameters. For lateral lying behaviour, a trend of interaction was found between diet and ∆THI (*p* = 0.086) (Table 3). This implies that the SF group had a steeper slope of lateral lying than the CF group with an increasing ∆THI (17 and 7% per ∆THI, respectively). Furthermore, with an increasing ∆THI, sitting (*p* = 0.084) and active behaviour (*p* = 0.079) tended to decrease, while inactive behaviour (*p* = 0.056) tended to increase.

#### 3.2.2. Correlations

When comparing T_rectal_ and T_skin_ during the second heat period, a low and negative correlation coefficient of −0.19 (*p* = 0.323) was found. During the entire growing–finishing period, the correlation coefficient between T_rectal_ and T_skin_ was 0.12 (*p* = 0.004), while the correlation was much higher for T_skin_ and ambient temperature (0.48, *p* < 0.001).

#### 3.2.3. Performance Parameters

The ADFI, ADG, and feed conversion ratio between the CF and SF groups did not significantly differ in the starter or the grower phases (*p* > 0.05). Nevertheless, we observed that the ADFI of the SF group was numerically lower in the starter and grower phase than the CF group (starter: 1562 g/day vs. 1645 g/day; grower: 2472 g/day vs. 2518 g/day, respectively). Also, the ADG of the SF group was numerically lower than in the CF group for both phases (starter: 787 g/day vs. 856 g/day; grower: 882 g/day vs. 905 g/day, respectively) (Table S1).

**Figure 3:**
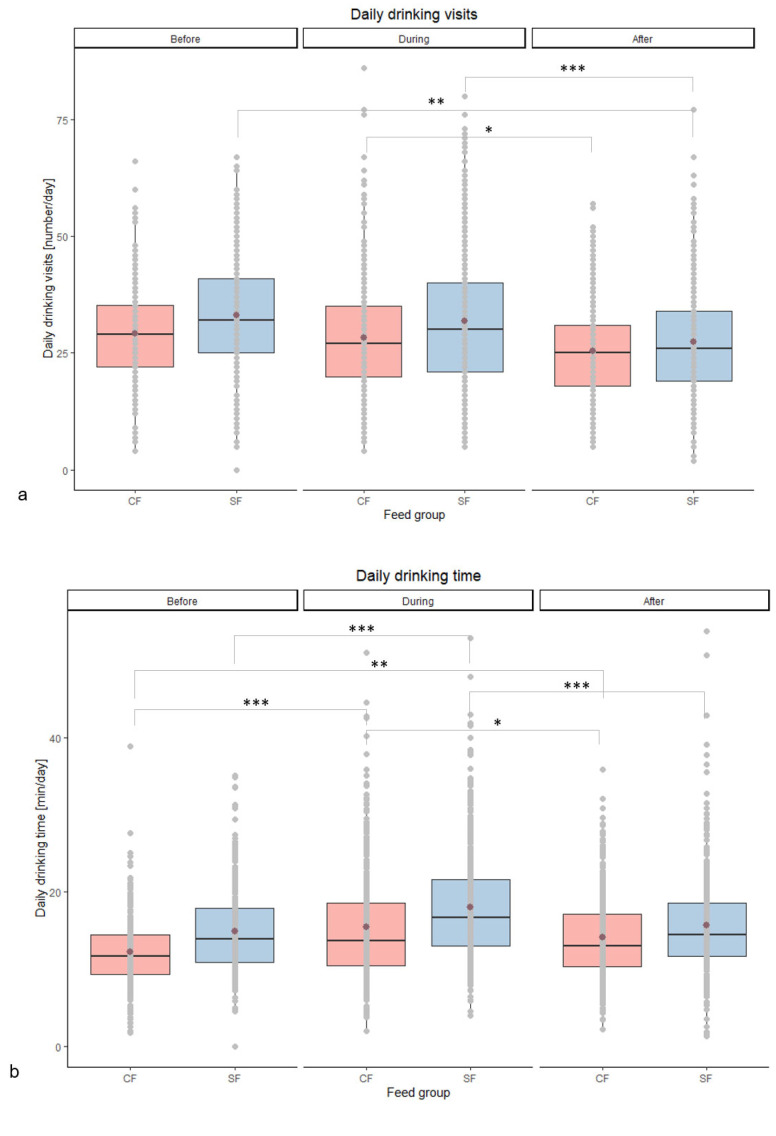

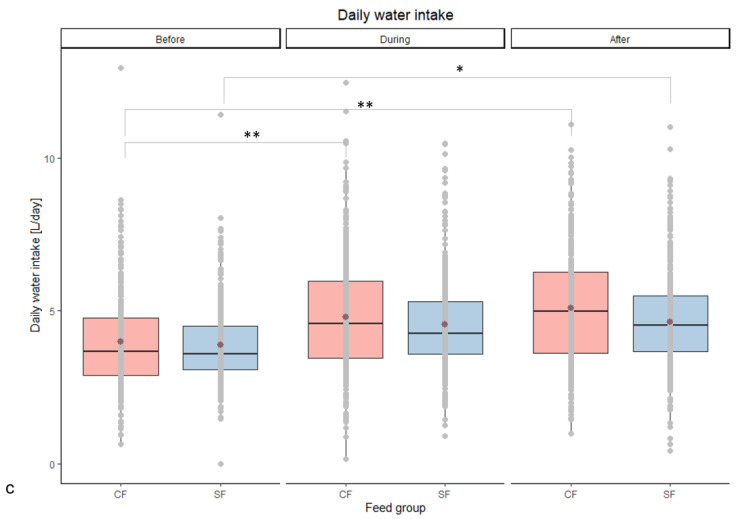
(**a**) Daily drinking visit [number/day], (**b**) daily drinking time [min/day], and (**c**) daily water intake [L/day] per diet group (control (CF) and summer feed (SF)) before, during, and after a period of heat load. Significant differences between diet groups and period of heat load are implemented by * (*p* ≤ 0.05), ** (*p* ≤ 0.01), or *** (*p* ≤ 0.001). (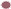 = mean of the parameter of the diet group.)

For daily drinking visits, daily drinking time, and ADWI, a significant interaction between diet x period of heat load (before, during, or after a higher heat load period) was found (*p* = 0.013, *p* = 0.03 and *p* = 0.013, respectively) (Table 4). During a period of higher heat load, the CF and SF groups showed a significantly higher daily drinking time compared to the period before (*p* < 0.001 and *p* < 0.001, respectively) and after a higher heat load (*p* = 0.048 and *p* < 0.001, respectively). For the CF as well as the SF group, the number of daily drinking visits dropped significantly after a higher heat load period (*p* = 0.024 and *p* < 0.001, respectively), as seen in the post hoc analysis (Figure 3). Furthermore, the CF group had a significantly higher ADWI during a period of higher heat load compared to the previous period (*p* = 0.009), while this was not the case for the SF group. Interestingly, the SF group generally had more drinking visits and longer drinking times than the CF group, while the total daily water intake of the SF group was lower (*p* > 0.05). No diet effect or diet x period of heat load interaction was found on ADFI. Nevertheless, there was an effect of heat load for both diet groups (*p* < 0.001). The ADFI of both diet groups was higher in the week after compared to the week before the higher heat load (Table 4).

### 3.3. Measurements in the Slaughterhouse

#### 3.3.1. Observations in the Lairage Area

No significant interaction between diet and observation time for heat stress score or skin lesions was found (Table S2). Also, no significant differences were found between the two diets for both parameters. However, for both diets, observation time showed a significant difference (*p* < 0.001) where the heat stress score was lower after an hour of lairage compared to the observation upon arrival at the slaughterhouse. There were no significant differences in panting, open mouth, drooling, and skin colour between the groups (*p* > 0.05).

#### 3.3.2. Carcass Traits and Meat Quality

Carcass parameters (warm carcass weight, cold carcass weight, carcass lean meat content, fat thickness, muscle thickness, dressing yield, and lean tissue growth) as well as meat quality (pH, water holding capacity, shear force, intramuscular fat content, and CIE-L*a*b* colour determinants) did not significantly differ between the CF and SF groups (*p* > 0.05). Furthermore, no significant differences were observed in the different sensory evaluation parameters (fried odour, ‘piggy’ odour, tenderness, juiciness, fried flavour, ‘piggy’ flavour, and acidic flavour) between the diet groups (*p* > 0.05) (Table S3).

## 4. Discussion

The aim of the present study was to evaluate an all-round strategy, i.e., concurrent addition of different additives throughout the entire growing–finishing period during the summer to mitigate the effects of heat stress. This approach was chosen for its practicality, namely applying a one-time adjustment during the entire summer growing–finishing period versus reacting only when heat load increased. Moreover, concurrent supplementation of the various antioxidants and osmolytes over the entire period represented the maximum potential of this strategy on all evaluated variables. In our study, the SF group showed no need to increase water intake during a higher heat load.

Despite relatively limited high heat load conditions, in this study, there was an increase in respiration rate. Pigs primarily depend on evaporative heat loss, and an elevated respiration rate is therefore the first physiological adaptation to the effects of heat stress [44]. This change may be indicative of potential impacts on production outcomes, which are of economic importance. Since farmers need to monitor easily noticeable parameters that change in the early stages of heat stress, this non-invasive parameter can be conveniently tracked by them. Additionally, in this study, meat quality emerged also as an important parameter. Feed additives were also considered for their potential positive effects on meat quality, irrespective of the presence of heat stress, in comparison to the control feed.

### 4.1. Measurements at Farm Level

#### 4.1.1. Physiological Parameters and Behaviour

For both the CF and SF groups, RR and T_skin_ increased significantly with an increasing ∆THI (above 75) or daily THI, respectively, while this effect was not significant for T_rectal_. These results indicate that RR and T_skin_ may be more sensitive parameters to assess HS than T_rectal_. This finding also reinforces that RR is the first physiological change to HS [8]. It is possible that the THI was too low to reach the inflexion point of T_rectal_ [8].

The effect of a higher heat load on physiological parameters did not differ between the two diets despite the anticipated effects based on literature. For example, Liu et al. [30] found a less steep increase in T_rectal_ during a heat load period of 8 days when feeding an organic selenium supplementation (1.0 ppm). Furthermore, Gabler et al. [45] found a decrease in RR in pigs fed a betaine-supplemented feed. Chauhan et al. [46] also found a decreased RR and T_rectal_ in sheep with vitamin E and selenium supplementation, and Attia et al. [47] found a decreased T_rectal_ in betaine-fed growing chickens. In agreement with our study, no effects of vitamin E and organic selenium on T_rectal_ could be demonstrated when the high heat load period only lasted 2 days [32]. The heat load in our study may have been too mild and/or too short to observe possible beneficial effects of the additives. It should be noted that HS severity and duration differs among studies which complicates a comparison of study results. In addition, little is known about the combined effect of the different additives. One study that included the simultaneous combination of selenium, vitamin E, and betaine in sows under HS did not find alterations in RR [31]. A study that combined selenium, vitamin E, and betaine in a diet for broilers found that the reduction in RR during HS due to betaine supplementation was less pronounced when selenium and vitamin E were also added [48]. These results were confirmed in a second study by Shakeri et al. [49], who had found a trend in a decreasing T_rectal_ in broilers when betaine was supplemented but not under concurrent supplementation of betaine, selenium, and vitamin E. The same study noted that for RR, a significant decrease was found when supplementing betaine that did not decrease further when selenium and vitamin E were supplemented in addition to betaine. This indicates a need for further research to evaluate the effects of concurrent supplementation of these nutrients in growing–finishing pigs during more extreme heat loads.

It is well-known that pigs increase their lateral lying behaviour when the heat load increases. When pigs lie on their side, the contact area with the cooler floor increases [9]. These findings were also found in the present study. However, the increase in lateral lying behaviour with an increasing ∆THI also differed between diets, as indicated by the diet × ∆THI interaction. Pigs fed SF increased their lateral lying behaviour by 10% more than the CF group with an increasing THI. Other behavioural parameters were not significantly affected by the dietary treatments.

#### 4.1.2. Correlations

Rectal temperature and RR are considered good parameters to evaluate HS at the animal level. These parameters are time-consuming to measure, however. Skin temperature could be an interesting non-invasive proxy for T_rectal_ and an indicator for HS in practice. Brown-Brandl et al. [39] state that thermal images can be a tool to indicate thermal comfort. However, the correlations between the T_rectal_ and T_skin_ of the flank indicates that T_skin_ is not reliable for this purpose, especially because the correlation coefficient during the entire growing–finishing period was positive, while this was a negative coefficient when examining only the data of the second and highest heat peak. The poor correlation between the T_rectal_ and T_skin_ of the flank was also found by Schmidt et al. [50], who observed that the surface temperature of a sow’s thigh obtained with an infrared camera was not in close agreement with T_rectal_. In addition, the results of Dewulf et al. [51] showed that T_skin_ and T_rectal_ had a linear relationship (*p* < 0.01), with a minimal slope of 0.044 °C increasing in T_rectal_ with an increase of 1 °C in T_skin_, but that T_skin_ cannot replace or predict T_rectal_. A relatively strong correlation was found between T_skin_ and ambient temperature, reinforcing the idea that T_skin_ may not be a reliable physiological parameter. This indicates that T_skin_ of the flank with infrared technology cannot replace the measurement of T_rectal_.

#### 4.1.3. Performance Parameters

Performance results did not show significant differences, but the SF group had numerically lower values for ADFI and ADG during the starter and grower phases. These numerical differences are relevant and are difficult to clarify. The flavour of certain additives may decrease ADFI with a resulting decrease in ADG. A review study on the function of betaine in pigs summarised that only 2 out of 41 studies showed significant adverse effects of betaine on ADFI in growing–finishing pigs [52,53]. A total of 15 studies found no differences, while all the other studies showed positive results on performance parameters [20]. In recent studies, betaine did not affect performance parameters [54,55]. For selenium, a meta-analysis indicates that selenium supplementation increases ADG and feed efficiency [56]. Based on the literature, it seems unlikely that selenium or betaine would negatively influence ADFI. Another explanation may be related to the number of pigs per feeder. In one of the two pens of the CF group (CF1), three animals had to be excluded from the trial at the beginning of the growing–finishing period. Therefore, the feed competition at the Nedap feeding system was probably lower due to reduced stocking density, which may have led to a higher ADFI of the individuals in CF1 and consequently a higher mean ADFI of the CF group. This was also found by Hyun and Ellis [57], where feed intake and growth rates were lower for groups with 12 growing–finishing pigs in the starter phase as compared to groups of 2, 4, 6, or 8 pigs, while this was not the case for growing–finishing pigs in the grower phase [58]. As the numerical difference between the CF and SF groups was more pronounced in the starter phase, the stocking density and/or feed competition may partly explain these findings. The diet composition might also contribute to a lower ADFI. Despite the efforts to maintain constant values, there are some small, unintended differences in the analysed diet composition. Typically, slight increases in crude protein content do not impact ADFI [59], but an increase in crude fibre may slightly reduce ADFI [60,61]. However, this seems unlikely, as the percentage increases in diet composition in SF were rather small.

The significant interactions in diet × period for daily drinking visits, daily drinking time, and ADWI imply that animals on SF behave differently during a higher heat load period. Water intake during a period of elevated THI increased significantly in the CF group while it was not changed in the SF group. Regardless of the treatment, all pigs suffered from a higher heat load, as reflected by the significant increase in RR (and T_skin_.) Previous studies indicate that pigs normally increase their ADWI during a period of high heat load [8]. Nevertheless, as the SF group showed no significant change in ADWI during a heat period, it may indicate that the SF pigs were less affected by the higher heat load than the CF group. To our knowledge, no study has yet shown an effect of dietary selenium or betaine addition on the water intake of growing–finishing pigs subjected to heat load. Because betaine functions as an osmoprotectant [20], also called compatible osmolyte [62], it can increase the water-binding capacity of the intestinal cells [63] and protect cell components from denaturisation due to high ionic strength [64]. This may result in a lower need to increase water intake during periods of more intense heat. Noticeably, the SF group had an overall higher number of drinking visits and spent more drinking time than the CF group, while their overall daily water intake was lower. This can be explained by the relatively higher average flow rate in the CF group (0.812 L/min) compared to the SF group (0.716 L/min).

The ADFI between the diet groups before, during, and after a heat load period showed no significant differences. Surprisingly, the ADFI for both groups increased significantly during a higher heat load period compared with the period before. Usually, a decrease in feed intake is expected during a heat load period [10,11,12], as this is one of the primary mechanisms in animals to reduce heat production [8]. According to Huynh, Aarnink, Verstegen, et al. [8], the inflexion point where voluntary feed intake starts to decrease is situated around 25.5 °C at a relative humidity of 65% (average relative humidity in this trial was 62%), which corresponds to a THI of 74 according to the formula of Lucas et al. [35]. During our trial, the average THI during the three heat peaks was higher (75.4) and therefore a decrease in ADFI can be expected. Possibly other factors such as stocking density played a role. The stocking density in our study (1.27 m^2^/pig) was lower than in the study of Huynh, Aarnink, Verstegen, et al. [8](1.1 m^2^/pig). This may lower the HS effects and/or increase the threshold value for a decreased ADFI. Because the period of a higher heat load is associated with the week of the growing–finishing period, the increasing ADFI over the three periods can also be regarded as an age effect because feed intake is correlated with live weight [65,66].

### 4.2. Measurements in the Slaughterhouse

#### 4.2.1. Observations in the Lairage Area

The summer feed had no effect on the heat stress score in the lairage area, but it should be noted that the pigs were not subject to HS during lairage, as the THI was 60.6 at the time [35,36,37,38]. The mean heat stress score after arrival in the lairage was only around 27, which is relatively low on a tagged visual analogue scale of 150. When staying in the lairage the heat stress score dropped further regardless of the diet group, which may indicate that the waiting time of one hour in the lairage is sufficient to have a reduction in overall stress.

#### 4.2.2. Carcass Traits and Meat Quality

No significant differences were found between diet groups for carcass traits and meat quality, which is in contrast to most studies of betaine on carcass traits. Two review studies stated that betaine supplementation increases lean meat content and decreases carcass fat content [20,21]. Furthermore, a study on the supplementation of vitamin E and selenium (and soy isoflavone) found a decreased back fat thickness at the last rib, an increased yellowness b* value, and decreased drip loss [67]. Also, supplementation of vitamin E and vitamin C may positively affect pH and drip loss [22]. In addition, vitamin E has been found to increase water-holding capacity and improve the colour of the loin [23,24,25,26]. Selenium, on the other hand, would contribute very little to meat quality [23].

This trial had certain weaknesses. The main objective was to investigate the different feed additives during periods of high heat load during a naturally warm summer. During the summer of the trial, there were no naturally occurring heat waves and the maximum daily THI remained relatively low throughout the trial. The stocking density was also lower than field conditions on a conventional farm. The pigs therefore had more space to cope with HS according to conductive and radiative heat transfer. In addition, although most parameters were measured at the individual level, statistically, the number of repetitions per diet treatment was only two due to the practical limitation that only one diet could be supplied per pen.

## 5. Conclusions

Dietary supplementation of betaine, selenomethionine, vitamin E, and vitamin C did not significantly alter the physiological and performance parameters of growing–finishing pigs raised under the tested summer conditions. Carcass and meat quality also showed no significant differences between the two diets. Pigs in the summer feed group had no altered water intake during the warm periods, which may indicate that they are better able to maintain homeostasis and/or suffer less from HS. Future research should focus on the effect of various additives in different proportions at higher heat loads.

## Figures and Tables

**Table 1:** Ingredients and analysed chemical composition of the control (CF) and summer feed (SF) [%] of the starter and grower phases.

	Diet			
	Starter Phase (10–15 Weeks of Age)		Grower Phase (15–25 Weeks of Age)	
Ingredients and Composition	CF	SF	CF	SF
Ingredients				
Wheat [%]	31.094	31.365	36.902	36.386
Barley [%]	20.000	20.000	20.000	20.000
Maize [%]	5.000	5.000	-	-
Soybean meal (48% crude protein) [%]	13.096	13.339	6.500	6.500
Biscuits [%]	8.000	8.000	7.500	7.500
Corn flakes [%]	3.000	3.000	3.000	3.000
Wheat middlings [%]	0.000	0.000	3.700	3.606
Wheat gluten [%]	8.590	5.317	13.000	13.000
Palm oil [%]	1.636	1.831	0.900	0.900
Beet molasses [%]	2.000	2.000	2.700	2.641
Palm kernels [%]	2.000	2.000	0.835	0.835
Beet pulp [%]	1.500	3.085	1.500	1.500
Feed chalk [%]	1.472	1.392	1.474	1.403
Table salt [%] ^1^	0.473	0.165	0.543	0.167
DL-Methionine [%]	0.203	0.208	0.131	0.130
L-Valine [%]	0.052	0.055	0.005	0.000
L-Lysine (50%) [%]	0.799	0.792	0.715	0.700
Tryptophan (25%) [%]	0.167	0.172	0.046	0.038
vL-Threonine [%]	0.202	0.198	0.200	0.160
Betaine [%] ^2^	0.000	0.667	0.000	0.667
Vitamin E [%] ^3^	0.020	0.030	0.020	0.030
Vitamin C [%] ^4^	0.000	0.057	0.000	0.057
Sodium bicarbonate [%] ^1^	0.000	0.450	0.000	0.400
Magnesium oxide [%]	0.121	0.128	0.109	0.110
Monocalcium phosphate [%]	0.119	0.246	0.067	0.067
Organic acid mix	0.300	0.300	-	-
Premix CF [%] ^5^	0.150	0.000	0.150	0.000
Premix SF [%] ^6^	0.000	0.200	0.000	0.200
Phytase [%]	0.006	0.003	0.003	0.003
Analysed chemical composition				
Crude protein (N × 6.25) [%V]	16.4	16.0	15.3	15.7
Crude fat [%V]	4.9	5.1	4.6	4.6
Crude ash [%V]	5.2	5.2	5.5	5.0
Crude fibre [%V]	4.5	4.6	4.5	4.8
Water [%V]	10.4	10.9	10.1	10.9
Lysine [g/kg] ^7^	10.6	10.5	9.0	8.9
NE [MJ/kg] ^7^	9.6	9.6	9.4	9.3

^1^ SF contained sodium bicarbonate instead of table salt. ^2^ SF contained 2000 mg/kg more betaine (anhydrate) than CF. ^3^ SF contained 100 mg/kg more vitamin E than CF. ^4^ SF contained 200 mg/kg more vitamin C than CF. ^5^ The premix of the CF contained 0.4 mg/kg of inorganic selenium. ^6^ The premix of the SF contained 0.3 mg/kg inorganic selenium and 0.1 mg/kg selenomethionine. ^7^ Calculated composition. Control feed; SF = Summer Feed; NE = Net Energy.

**Table 2:** Definitions of active and inactive behaviour of growing–finishing pigs.

Animal Behaviour		
**Active ^1^**	Standing	Body supported by three or more legs and with head raised
	Moving	Walking or running, body supported by three or more legs, position change possible and head held high.
	Exploring	Sniffing the floor and feeder, interacting with materials or pen mates
	Sitting	One or two front legs support the body, with hindquarters touching the ground.
**Inactive**	Sternal lying	The pig lies on its sternum with its head high or down and 0 or 2 legs are extended
	Semi-sternal lying	The pig lies on its sternum with its head high or down and 2 legs extended, or the pig lies (half) on its side with only two legs extended.
	Lateral lying	The pig lies entirely on its side with four legs extended.

^1^ Drinking and/or eating was also registered but not included in the model, as the feeder and drinking nipple were occupied at almost every behavioural assessment.

**Table 3:** Effect of diet (control (CF) and summer feed (SF)), ∆THI (temperature–humidity index), and its interactions on physiological parameters and animal behaviour of growing–finishing pigs housed during the summer season. The slope indicates the increase/decrease in the parameter when THI increases with 1 value from a baseline THI of 75.

	Diet						
Parameters	Baseline at THI = 75		Slope ^1^ per ∆THI or THI ^2^		*p*-Value		
	CF	SF	CF	SF	Diet	∆THI	Diet × ∆THI
**Physiological parameters**							
Respiration rate [breaths/min]	46.3	45.7	34.5	34.5	0.904	<0.001	n.s.
Rectal temperature [°C]	39.6	39.6	0.129	0.129	0.869	0.216	n.s.
Skin temperature ^2^ [°C]	35.1	35.2	0.294	0.294	0.875	<0.001	n.s.
**Animal behaviour [%]**							
Active behaviour	43	43	−14	−14	1.000	0.079	n.s.
Standing	11	11	−5	−5	0.975	0.186	n.s.
Exploring	25	23	−5	−5	0.669	0.108	n.s.
Sitting	7	8	−4	−4	0.204	0.084	n.s.
**Inactive behaviour [%]**	47	49	15	15	0.649	0.056	n.s.
Sternal lying	37	35	0	0	0.659	0.965	n.s.
Semi-sternal lying	3	5	4	4	0.334	0.281	n.s.
Lateral lying	8	8	7	17	0.971	0.298	0.086

n.s. = no significant interaction; CF = control feed; SF = summer Feed; THI = temperature–humidity index. ^1^ The slope increase of the two treatments is the same if no interaction or other significant difference was found between treatments. ^2^ Skin temperature according to THI (linear) instead of ∆THI due to its linear progression.

**Table 4:** Effect of diet (control (CF) and summer feed (SF)), the period of heat load (before, during, and after), and their interactions on drinking parameters and daily feed intake of growing–finishing pigs housed during the summer season. Each period (before, during, and after a heat peak) consists of 16 days.

Parameter	Period of Heat Load	Diet		SEM	*p*-Value		
		CF	SF		Diet	Period	Diet × Period
Daily drinking visits [number/day]	Before	29 ^abcd^	33 ^bd^	0.43	0.150	0.008	0.013
	During	28 ^cd^	32 ^bd^	0.45			
	After	26 ^ab^	27 ^ac^	0.37			
Daily drinking time [min/day]	Before	12 ^ab^	15 ^ace^	0.20	0.099	<0.001	0.036
	During	15 ^ef^	18 ^bdf^	0.24			
	After	14 ^cd^	16 ^ace^	0.20			
Daily water intake [L/day]	Before	4.0 ^ac^	3.9 ^ab^	0.06	0.730	<0.001	0.013
	During	4.8 ^bd^	4.6 ^abcd^	0.05			
	After	5.1 ^bd^	4.6 ^cd^	0.06			
Daily feed intake [g/day]	Before	2088 ^ac^	2007 ^ab^	25.7	0.503	<0.001	n.s.
	During	2144 ^abcd^	2085 ^abcd^	18.5			
	After	2296 ^bd^	2234 ^cd^	19.8			

n.s. = non-significant interaction; CF = control feed; SF = summer feed. ^a–f^ Values within a row (daily drinking visits, daily drinking time, daily water intake, and daily feed intake) with different superscripts differ significantly at *p* < 0.05.

## Data Availability

The analysed datasets from the current study are available from the corresponding author upon request.

## Supplementary Materials

The following supporting information can be downloaded at https://www.mdpi.com/article/10.3390/vetsci11030110/s1, Material and Methods S1, Table S1: Effect of diet (control (CF) and summer feed (SF)) per phase (starter (10–15 weeks of age) and grower phase (15–25 weeks of age)) on average daily feed intake, average daily gain, and feed conversion ratio of growing–finishing pigs housed during the summer season; Table S2: Effect of diet (control (CF) and summer feed (SF)), observation time point in the lairage area (upon arrival and one hour after), and its interactions on heat stress score and skin lesions of growing–finishing pigs housed during the summer season; Table S3: Effect of diet (control (CF) and summer feed (SF)) on carcass traits and meat quality (instrumental and sensory) of growing–finishing pigs housed during the summer season.
