# Supplementary material for: Effect of Simultaneous Dietary Supplementation of Betaine, Selenomethionine, and Vitamins E and C under Summer Conditions in Growing–Finishing Pigs
Source: Vet Sci. 2024 Mar 1;11(3):110. doi: 10.3390/vetsci11030110 (PMC10974906; doi:10.3390/vetsci11030110)
Supplement: Supplementary file 1 [file vetsci-11-00110-s001.zip › Supplementary File.pdf]

**Effect of simultaneous dietary supplementation of betaine, selenomethionine, and vitamins E and C under summer conditions in growing-finishing pigs**

L. De Prekel, D. Maes, A. Van den Broeke, B. Ampe, M. Aluwé

*Veterinary Sciences*

**Supplementary material and methods S1**

***Measurements in the slaughterhouse***

*Observations in the lairage area*

All pigs were transported to a commercial slaughterhouse at 25 weeks of age. Upon arrival at the slaughterhouse, the same selected reference pigs were observed in the lairage area. Heat stress level was assessed upon entry to lairage and again one hour later. Heat stress was scored according to a tagged visual analogue scale of 0-150. Presence of panting, open mouth and drooling were also observed (yes/no) at the time of heat stress scoring. Skin colour (pink, red or red-spotted) and skin lesions (on a scale from 0 to 4) were also assessed at the same time as HS scoring.

*Carcass traits*

Pigs were slaughtered by exsanguination after carbon dioxide stunning. The 'AutoFOM III™' system (Frontmatec, Denmark) was used to define different carcass traits, as described by Kowalski et al. (2020). Warm carcass weight, cold carcass weight, carcass lean meat content, fat thickness, muscle thickness, dressing yield and lean tissue growth were determined.

*Meat quality: instrumental*

At 35 minutes post-mortem, the initial pH (Type HI98163 electrode FC2323, Hanna Instruments®, Temse Belgium) of all 57 animals was measured in the *M. longissimus thoracis et lumborum* next to the 7$^{th}$ rib (starting from the rostral side of the carcass). About 22h after slaughter, loin samples of the left side of all test animals were collected in the slaughterhouse. Twenty-four hours post-mortem, the ultimate pH was measured at the bottom, the middle and the top of the loin sample. Then the *M. longissimus thoracis et lumborum* was cut into meat slices and visible connective tissue and fat were removed. Water holding capacity was evaluated according to the gravimetric EZ-drip loss method of Christensen (2003), where two slices of 2.5cm were used. From each meat slice, a circular sample (Ø 25mm) between 9.5-10.5g was cut with a circular knife and weighed. Then, each sample was put into an EZ-drip loss container and stored for 48h at 4°C. The sample was weighed after 48h and the drip loss percentage calculated. Cooking loss and Warner-Bratzler (WB) shear force were measured according to the methods of Boccard et al. (1981) and Honikel (1998). Two slices of 2.5cm were put in a warm water bath of 75°C for 60 minutes and cooled down for a

minimum of 15 minutes by tap water. Then five cylindrical samples (Ø 1.27mm) per slice were cut parallel to the fibre direction and sheared perpendicular to the fibre direction using the Warner-Bratzler shear (Multitest 2.5-DV and AFG 250N, Mecmesin, UK). Afterwards, the average of all ten measurements per animal was calculated. Intramuscular fat content was measured according to the Bligh and Dyer method (Hanson J., 1963). First, two meat slices of 2.5cm were homogenised with a meat grinder and an ULTRA-TURRAX (ULTRA-TURRAX®, IKA®-Werke GmbH & Co. KG, Germany). Then, 100g of the homogenised sample was put in the NIRS device (NIRS DS2500 L™, FOSS, Denmark).The CIE-L*a*b* colour determinants were measured by three repeated measurements on 2.5cm meat slices with reflection spectroscopy (Miniscan® EZ 4500 L, Hunterlab, USA) after 30 minutes of blooming at 9 °C. Hue (colour vividness) and chroma (colour saturation index) were calculated according to the following formulas (AMSA, 2012):

$$Hue = \tan^{-1}\left(\frac{b*}{a*}\right)$$

$$Chroma = \sqrt[2]{a*^2 + b*^2}$$

With L*: lightness, a*:redness and b*:yellowness

When collecting the loin samples for meat quality, two carcasses were misplaced and were not included in the dataset.

*Meat quality: sensory*

Sensory evaluation of the loin samples was performed by a trained panel. Five sessions were organised in which 11 samples were served per session, with five or six samples per diet group. Each sample was scored by six trained panellists (according to Arildsen Jakobsen et al. (2014)) on different characteristics (fried odour, piggy odour, softness, juiciness, fried flavour, piggy flavour and acidic flavour) by a visual analogue scoring from 0-100. Between each sample, the panellists cleansed their palate by drinking water and eating an unsalted cracker. Each sample (slices of 2.0cm) was grilled on a clean pan (Tefal Ultra Compact Comfort GC3060, Tefal, Rumilly, France) to an inner temperature of 72°C. No herbs, salt or fat were added. Each slice was divided into six pieces and covered by a plastic cup to retain the aroma.

## ***Statistical analysis***

The effect of diet and observation time point during lairage (upon arrival and one hour after) on the heat stress score and skin lesions was evaluated using a linear mixed model with the pen as a random variable. The effect of diet on the different parameters of carcass traits, instrumental meat quality and sensory evaluations was also evaluated by a linear mixed model with pen as random variable for carcass traits and instrumental meat quality and panel time as random variable for the sensory meat quality. A post

hoc was performed when significant differences were found. The pen was considered as experimental unit.

## References


AMSA. (2012). AMSA Meat Color Measurement Guidelines. *Meat and Muscle Biology*. https://doi.org/10.22175/mmb.12473

Arildsen Jakobsen, L. M., Vuholm, S., Aaslyng, M. D., Kristensen, M., Sørensen, K. V., Raben, A., & Kehlet, U. (2014). Sensory characteristics and consumer liking of sausages with 10% fat and added rye or wheat bran. *Food Science & Nutrition*, *2*(5), 534–546. https://doi.org/10.1002/FSN3.126

Boccard, R., Buchter, L., Casteels, E., Cosentino, E., Dransfield, E., Hood, D. E., Joseph, R. L., MacDougall, D. B., Rhodes, D. N., Schön, I., Tinbergen, B. J., & Touraille, C. (1981). Procedures for measuring meat quality characteristics in beef production experiments. Report of a working group in the commission of the European communities' (CEC) beef production research programme. *Livestock Production Science*, *8*(5), 385–397. https://doi.org/10.1016/0301-6226(81)90061-0

Christensen, L. B. (2003). Drip loss sampling in porcine m. longissimus dorsi. *Meat Science*, *63*(4), 469–477. https://doi.org/10.1016/S0309-1740(02)00106-7

Hanson J., S. W. F. & O. (1963). Application of the Bligh and Dyer method of lipid extraction to tissue homogenates. *Biochem. J., 89 : 101-1O2*.

Honikel, K. O. (1998). Reference methods for the assessment of physical characteristics of meat. *Meat Science*, *49*(4), 447–457. https://doi.org/10.1016/S0309-1740(98)00034-5

Kowalski, E., Vossen, E., Millet, S., Ampe, B., Callens, B., van Royen, G., de Smet, S., & Aluwé, M. (2020). Performance and carcass, loin and ham quality in crossbreds from three terminal sire lines. *Meat Science*, *167*. https://doi.org/10.1016/j.meatsci.2020.108158

## Supplementary Tables

*Table S1: Effect of diet (control- (CF) and summer feed (SF)) per phase (starter- (10-15 weeks of age) and grower phase (15-25 weeks of age)) on average daily feed intake, average daily gain and feed conversion ratio of growing-finishing pigs housed during the summer season*

| Parameter | Phase | Diet | | SEM | P-value |
|---|---|---|---|---|---|
| | | CF | SF | | Diet |
| Average daily feed intake [g/day] | Starter | 1645 | 1562 | 32.1 | 0.328 |
| | Grower | 2518 | 2472 | 48.6 | 0.690 |
| Average daily gain [g/day] | Starter | 856 | 787 | 16.8 | 0.165 |
| | Grower | 905 | 882 | 13.3 | 0.478 |
| Feed conversion ratio | Starter | 1.92 | 1.99 | 0.01 | 0.310 |
| | Grower | 2.78 | 2.81 | 0.03 | 0.446 |

CF = Control feed; SF = Summer Feed

*Table S2: Effect of diet (control- (CF) and summer feed (SF)), observation time point in the lairage area (upon arrival and one hour after) and its interactions on heat stress score and skin lesions of growing-finishing pigs housed during the summer season*

| Parameter | Observation time point | Diet | | SEM | P-value | | |
|---|---|---|---|---|---|---|---|
| | | CF | SF | | Diet | Observation time point | Diet x observation time point |
| Heat stress score | upon arrival | 27.7[a] | 26.2[a] | 1.63 | 0.587 | <0.001 | n.s. |
| | one hour after arrival | 19.0[b] | 17.5[b] | 1.58 | | | |
| Skin lesions | upon arrival | 0.4 | 0.4 | 0.11 | 0.913 | 0.255 | n.s. |
| | one hour after arrival | 0.6 | 0.6 | 0.12 | | | |

n.s. = non-significant interaction; CF = Control feed; SF = Summer Feed

[a- b] Values within a row with different superscripts differ significantly at $P < 0.05$

*Table S3: Effect of diet (control (CF) and summer feed (SF)) on carcass traits and meat quality (instrumental and sensory) of growing-finishing pigs housed during the summer season*

| Parameters | Diet | | SEM | P-value |
|---|---|---|---|---|
| | CF | SF | | Diet |
| Carcass traits | | | | |
| Fasted weight [kg] | 119.0 | 116.0 | 1.49 | 0.476 |
| Warm carcass [kg] | 97.2 | 94.6 | 1.24 | 0.409 |
| Cold carcass [kg] | 95.3 | 92.8 | 1.21 | 0.409 |
| Lean meat content [%] | 63.8 | 63.4 | 0.38 | 0.637 |
| Fat thickness [mm] | 8.4 | 8.6 | 0.37 | 0.787 |
| Muscle thickness [mm] | 67.6 | 65.5 | 0.66 | 0.243 |
| Dressing yield [%] | 80.6 | 79.8 | 0.20 | 0.503 |
| Lean tissue gain [g/day] | 449.0 | 429.0 | 4.90 | 0.172 |
| Meat quality: instrumental | | | | |
| $pH_i$ | 6.5 | 6.5 | 0.04 | 0.545 |
| $pH_{24}$ | 5.6 | 5.6 | 0.01 | 0.346 |
| Drip loss [%] | 7.3 | 6.3 | 0.26 | 0.439 |
| Cooking loss [%] | 30.6 | 28.3 | 0.29 | 0.217 |
| Shear force [N] | 30.4 | 32.4 | 0.91 | 0.553 |
| Intramuscular fat [%] | 1.9 | 2.1 | 0.09 | 0.363 |
| Lightness L* | 57.1 | 57.4 | 0.27 | 0.603 |
| Redness a* | 6.5 | 6.2 | 0.08 | 0.332 |
| Yellowness b* | 14.9 | 15.1 | 0.07 | 0.480 |
| Hue | 66.6 | 67.6 | 0.25 | 0.172 |
| Chroma | 16.3 | 16.3 | 0.08 | 0.975 |
| Meat quality: sensory | | | | |
| Fried odour | 30 | 28 | 2.50 | 0.681 |
| 'Piggy' odour | 18 | 16 | 0.94 | 0.298 |
| Tenderness | 49 | 44 | 2.13 | 0.218 |
| Juiciness | 45 | 48 | 1.25 | 0.253 |
| Fried flavour | 22 | 20 | 2.01 | 0.550 |
| 'Piggy' flavour | 25 | 26 | 0.84 | 0.541 |
| Acidic flavour | 11 | 12 | 0.78 | 0.611 |

CF = Control feed; SF = Summer Feed
